# Factors Affecting Surface Plasmon Coupling of Quantum Wells in Nitride-Based LEDs: A Review of the Recent Advances

**DOI:** 10.3390/nano11051132

**Published:** 2021-04-27

**Authors:** Muhammad Farooq Saleem, Yi Peng, Kai Xiao, Huilu Yao, Yukun Wang, Wenhong Sun

**Affiliations:** 1 Research Center for Optoelectronic Materials and Devices, School of Physical Science & Technology, Guangxi University, Nanning 530004, China; farooq@mail.ustc.edu.cn (M.F.S.); 1907401031@st.gxu.edu.cn (Y.P.); 2007401020@st.gxu.edu.cn (K.X.); huiluy@gmail.com (H.Y.); 20180116@gxu.edu.cn (Y.W.); 2 Guangxi Key Laboratory for Relativistic Astrophysics, School of Physical Science & Technology, Guangxi University, Nanning 530004, China; 3 Guangxi Key Laboratory of Processing for Non-Ferrous Metallic and Featured Materials, Guangxi University, Nanning 530004, China

**Keywords:** LEDs, GaN, surface plasmon, quantum-well, nanoparticles

## Abstract

Surface plasmon (SP)-enhanced quantum-well (QW) LEDs have proved their potential in replacing conventional lighting devices for their high-performance capabilities in ultraviolet (UV), blue and green spectral ranges. The SP-enhanced QW-LEDs have applications in light emission enhancement, light polarization, color conversion, and speed modulation. The electric field of the plasmonic mode of a metal couples with the exciton energy of QWs in resonance results in efficiency enhancement to several folds. The strength of the SP–QW coupling is mainly influenced by the type of metal used for SP enhancement, the metal nanostructure geometry, and the penetration depth of the SP fringing field in the p-GaN. The use of an appropriate dielectric interlayer between the metal and the p-GaN allows further control over SP resonance with QW emission wavelength. The penetration depth defines the p-GaN thickness and the QW period number for effective SP–QW coupling. The optimization of these parameters is key to achieve high efficiencies in SP-enhanced QW-LEDs for various applications. This review explains the SP enhancement mechanism and the key challenges facing the SP enhancement of QW-LEDs. The main factors that affect the SP–QW coupling have been explained in detail based on recent reports devoted to this field.

## 1. Introduction

The surface charge density oscillations that exist at a metal–dielectric interface are known as surface plasmons. The study that deals with the control and utilization of these plasma oscillations is known as plasmonics [1]. These oscillations carry non-radiative electromagnetic surface waves along the interface. SPs at the metal surface can interact with light of the same frequency and wave vector, thereby generating a fluctuating electric field at the interface. The phenomenon is known as surface plasmon resonance (SPR). The SPs generate giant electromagnetic fields that can support light emission, absorption, and scattering enhancement in various materials and devices. The phenomenon is used in many spectroscopic and device applications such as surface-enhanced Raman scattering, surface plasmon resonance spectroscopy, LEDs, etc. Thin films, random nanostructures, periodic nanostructures, and gratings of different metals are used to excite SPs. The SPR intensities and frequencies depend on various factors such as morphology, arrangement, and dielectric environment of the metal nanostructures. Localized surface plasmons (LSPs) are generated by using metal nanoparticles (NPs). The propagating SPs known as surface plasmon polaritons (SPPs) are generated at a relatively smooth and continuous metal–dielectric interface. The periodic metal structures possess even more exciting properties such as plasmonic band gaps and light control beyond the diffraction limit [2]. For the applications in the visible range of the electromagnetic spectrum, Au and Ag are found to be the most promising plasmonic materials for their large negative real part of the relative permittivity and small imaginary part of permittivity in the visible range [3].

Light emission enhancement in LEDs is one of the most important applications of plasmonics due to the growing need for energy-efficient devices. LEDs have attracted much attention since the achievement of light emission in the visible region of the electromagnetic spectrum [4]. The high brightness and low energy consumption compared with conventional lighting devices are the most favorable characteristics of LEDs [5,6,7]. SP enhancement has been successfully applied to QW-LEDs as well as organic LEDs. The SP enhancement of QW-LEDs was theoretically proposed by E. Yablonovitch and experimentally demonstrated by K. Okamoto [8,9,10]. Since then, SP-enhanced QW-LEDs of various device structures and materials have been proposed. The high-efficiency LEDs using this approach have the potential to replace the conventional lighting devices, but far more research is needed to realize high efficiency and cost-effective LEDs for practical applications. SPs serve various purposes in LEDs such as the enhancement of the emission efficiency, increasing the light extraction efficiency, the polarization of the emitted light, speed modulation, and increasing the color conversion efficiency [1,2,3,4,5,6,7,8,9,10,11,12,13,14,15,16].

The nitride-based LEDs contain QWs sandwiched between the p and n-type GaN layers. The QWs contain excitons that decay as a result of photo or electrical pumping of an LED. To achieve the SP enhancement in QW-LEDs, the plasmonic metal structures are formed on the p-GaN surface layer. SP enhancement has been demonstrated in both the single quantum well (SQW) and multiple quantum well (MQW) LEDs. The number of QWs affects the strength of SP–QW coupling and emission efficiency [17]. MQWs, when simultaneously coupled with the same SP resonance mode of metal nanostructures, can result in superradiance. The near field SP resonance coupling of metal nanostructures and QWs has shown strong dependence on the distance between the metal and the QWs. The appropriate coupling distance between the QW and the metal can enhance the SP resonance. A QW, when placed within the penetration depth of an SP fringing field, can experience a strong electrodynamical coupling to the SP mode [18]. Above a critical distance between the metal and QW, the plasmonic oscillations are reported to weaken as the SP fringing field declines above a critical penetration depth. At the same time, the p-GaN layer that defines the distance between the QW and metal should be kept sufficiently thick to maintain the p–n junction and preserve the electrical properties of an LED. A thick p-GaN layer of usually more than 150 nm is necessary for effective current spreading. On the other hand, the SP electric field, being evanescent, decays within the thickness of below a hundred nm. For the successful SP–QW coupling, the p-type layer should be kept sufficiently thin. The possible workarounds such as metal protrusions, embedded metal NPs, and the deep metal grating structures are effective but costly and complicated [19,20,21,22,23,24]. This review explains the basic SP–QW coupling mechanism, key challenges and the most important factors affecting the SP enhancement in QW-LEDs based on recent reports devoted to this field.

## 2. SP Enhancement Mechanism in Nitride-Based QW-LEDs

**Figure 1:**
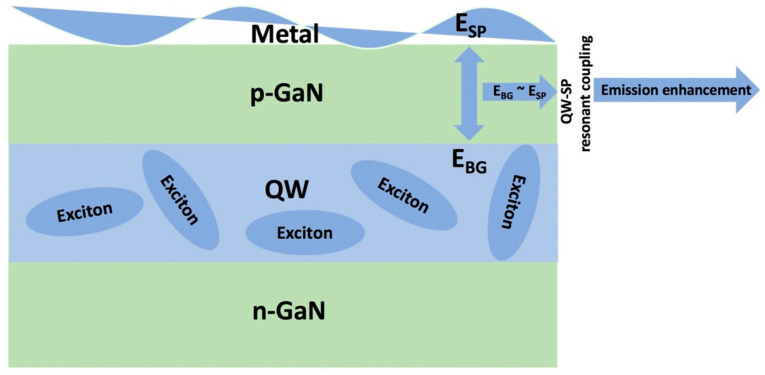
Schematic representation of SP–QW coupling mechanism.

The nitride-based QW-LEDs are fabricated by sandwiching a QW layer between p and n-type GaN layers. AlGaN or InGaN can be used as QW materials for the development of nitride-based LEDs depending on their applications in the UV or visible ranges. The QWs contain excitons that can decay both radiatively and non-radiatively. The radiative recombination that is the desirable property of a QW in an LED, can be enhanced by SP–QW coupling. The exciton–SP coupling can be achieved by using metal films, NPs, or gratings in the vicinity of QWs that can enhance the emission efficiency of an LED. The SPs of high momentum lose their energies through scattering and couple to radiated light, resulting in efficient SP–QW coupling [25]. The strong SP–QW coupling is achieved when the QW emission energy is close to the SP resonance wavelength of the metal nanostructures. Such an alignment of the SP resonance wavelength and QW emission wavelength results in increased spontaneous emission. K. Okamoto et al. explained the phenomenon by using a schematic diagram similar to the one shown in Figure 1 [26]. They proposed that the excitons are formed in QWs as a result of photo or electrical pumping. The radiative recombination in such devices can be enhanced by using a metal layer in the near-field of QWs that results in a fluctuating electric field. The high density of states of SPs in metal produces high electromagnetic fields. The spontaneous emission in QWs increases with the local density of states and the strength of the electromagnetic field of SPs, known as the Purcell effect [27]. The Purcell effect is associated with the change in carrier lifetime in the presence of SPs in LEDs. When the bandgap energy (E_BG_) of the QW layer is in close resonance with the vibration energy (E_SP_) of the fluctuating electric field of the SPs on the metal interface, the QW energy efficiently transfers to the SPs, known as SP–QW coupling. Strong SP–QW coupling can be achieved by controlling the geometry of NPs that in turn gives control over the E_SP_ [28,29]. The rate of exciton recombination increases with the rate of SP–QW coupling. The exciton–SP coupling process is faster than radiative and non-radiative processes in an LED due to the large density of states of SP modes. The increased density of states of SPs results in a fast decay rate due to the increased overlap of electron and hole wave functions [30].

## 3. Key Performance Parameters

The emission enhancement in an SP-coupled LED can be estimated either by photo or electrical pumping, known as photoluminescence (PL) and electroluminescence (EL), respectively. LEDs work by electrical pumping in practical device applications, while PL is widely used to roughly estimate the emission efficiencies of LEDs. The external quantum efficiency (EQE) of a QW-LED depends on its injection efficiency, internal quantum efficiency (IQE), and light extraction efficiency (LEE). The IQE is defined as the ratio of radiative recombination rate to the sum of radiative and non-radiative recombination rates of QW-LEDs. By assuming the non-radiative recombination rate to be negligible at 10 K, the IQE is evaluated by taking the ratio of integrated PL intensities at 300 K to that of 10 K [17]. The energy can be radiated out and/or dissipated in the system that is governed by the LEE. It defines the probability of light escaping out of an LED.

## 4. State-of-the-Art and Challenges

LEDs are known to give far less EL enhancement compared with PL [31]. The injection efficiency can be enhanced by improving material quality and increasing doping densities. Improvement of the IQE and LEE is the major challenge in EQE enhancement [32]. The factors that limit the IQE include the nonradiative recombination processes, dislocations/defects, and separation of the electron-hole wave functions by spontaneous polarization and strain-induced piezoelectric polarization [33,34,35,36,37,38,39]. The LEE of an LED is limited by the severe total internal reflection due to the difference in refractive indices of the device surface and air. The difficulty of light extraction from the opaque metal sides is another source of energy loss within the LEDs. The light trapped in the LEDs generates heat that reduces the life span of an LED. Despite the use of transparent and polished substrates, the improvement of LEE above 30% remains a challenge.

The reduction in radiative recombination lifetime, increase in the rate of radiative recombination, and improvement of LEE are the main challenges that cannot be achieved by conventional approaches [32]. The increased non-radiative recombination that results in poor IQE can be partly converted to radiative recombination by SP coupling. Exciton–SP coupling increases IQE by increasing spontaneous emission and reducing the non-radiative recombination [40,41,42,43]. The energy of SPs can be dissipated within the metal in the form of nonradiative losses via interband absorption of SPs [44]. To avoid the absorption losses and increasing the scattering of SPs of high momentum, the rough metal surfaces, metal nanostructures, or gratings are used that give rise to LSPs. The LSPs of high momentum couple to radiated light by losing their energies through scattering, resulting in light emission enhancement [25]. The LSP energy radiates effectively without the need of a phase/momentum-matching condition [45]. The metal nanostructures should be carefully designed to cover the emission wavelength range of the QW-LEDs for strong resonance [17,19]. The size and periodicity of the metal NPs affect the scattering and diffraction of light [40,46,47]. The morphology of NPs affects the strength of LSP and light scattering efficiency for effective resonance coupling with the QW emission spectrum [20,45]. Both the LSPs and SPPs can be excited simultaneously by using metal gratings for enhanced coupling with QWs [48]. The NPs of various metals, sizes, shapes, and periodicity can be fabricated that allow for the tunability of LSP resonance frequency to match with the emission frequency of the LED. Conversely, the use of a continuous metal film gives poor control over SPP resonance [49]. The IQE and EL are enhanced by SPs excited in the metal, while LEE enhancement in such LEDs is governed by the scattering or mirror effect of the metal [50]. A metal used on the top of the p-GaN layer can change the propagation angle of the light confined in the traps. Thus, the probability of light scattering out of the device increases [51]. A dielectric layer of low refractive index (compared with that of p-GaN) between metal and GaN is found to blue-shift the LSP resonance wavelength, strengthen the SP–QW coupling and increase the LEE [52,53]. It allows control over the SP resonance wavelength of the metal nanostructures for strong resonance with QW emission wavelength [17,54].

The SP-coupled LEDs also suffer many efficiency limiting issues that require proper device engineering and designing. The poor current spreading in a thin p-GaN layer, for instance, is a serious limitation that is resolved by using a metal as a current spreading layer. The metal nanostructures on the top covered with the current spreading metal layer result in the shift of the SP resonance peak away from the blue–green region. A thick p-GaN layer with deeply etched nanostructures filled with metals can give SP enhancement without interacting with the current spreading layer. However, current leakage is a serious limitation of such a structure. Embedding metal NPs in the p-GaN layer close to the QW interface can be a possible workaround, but it has drawbacks such as high fabrication cost, the low planar density of metal NPs for guaranteeing high re-growth quality, and weak LSP resonance [21].

While the blue LEDs have been commercialized with an external quantum efficiency (EQE) higher than 80%, the green LEDs are yet facing the “green-gap” (low emission efficiency of green LEDs) problem [49]. The green LEDs are obtained by increasing the In composition in QWs. Increased In content supports green emission but limits the EQE [55,56]. The low temperature-grown QWs with a high In concentration result in increased defect density and polarization-induced high built-in electric field due to lattice mismatch. The large built-in electric field leads to energy band bending in QWs, forcing the electrons and holes to opposite ends. The electron-hole wave function overlap is reduced, resulting in the low probability of radiative recombination, known as the quantum confined stark effect (QCSE). The IQE in QW-LEDs is strongly dependent on the QCSE and exciton dynamics [31].

In blue LEDs, carrier localization occurs that is a favorable phenomenon as it can alleviate the QCSE. Exciton localization occurs when excitons in the QW layer confine into In-rich regions. The excitons bound to these regions are well protected against thermal loss and have high emission efficiencies in blue LEDs. In green LEDs, carrier localization introduces additional Auger recombination loss, resulting in the efficiency droop effect (EDE) [57,58,59,60]. EDE is the reduction in efficiency with the increase in injection current. The two possible mechanisms contributing to EDE include the defect-assisted Auger recombination and carrier overflow from the QWs. The reduction in defect density and strain relaxation are the possible solutions to these issues that can support radiative recombination [27]. A few other solutions have been employed, such as quantum dot structure, screening by doping, the design of the QW structure by bandgap engineering, pre-strained MQWs, and the use of semipolar or nonpolar substrates [61].

The EQE can also be increased by SP coupling of the green QW-LEDs [10,62,63]. SP enhancement is achieved in LEDs by limiting and eliminating several effects that limit the emission efficiencies such as EDE, QCSE, and leakage through ohmic contact. EDE and “green-gap” problems can be eliminated by reducing the nonradiative recombination channels through SP–exciton coupling in QWs [49,52]. In the SP-enhanced QWs, the excitons decay rapidly, leading to a fast decrease in exciton density. Localization is a slow process compared with spontaneous emission in the SP-enhanced QW-LEDs that results in the cancellation of localization and QCSE [64]. The carrier energy can be effectively transferred into the SP mode in SP-coupled LEDs, providing an alternative emission channel. Energy transfer leads to increased exciton recombination and a reduction in carrier density.

QWs with emission wavelengths in the UV range are fabricated by alloying AlN with GaN [65]. UV-LEDs based on AlGaN QWs that have many applications in sensing, phototherapy, biological detection, sterilization, optical catalysis, and air/water purification, also suffer from low EQE [66,67,68,69]. The increased concentration of Al in AlGaN QWs results in poor doping efficiencies and crystal quality that limit the IQE of such LEDs [68,70,71]. Both the transverse magnetic (TM) and transverse electric (TE) polarized emissions are produced in active layers of UV-LEDs where only the TE mode can escape the device structure [72]. UV absorption in p-GaN and TM-polarized emissions in active layers contributes to the poor LEE of AlGaN QW-LEDs [15]. The metals suitable for SP enhancement in the UV range are also limited. Consequently, the efficiency enhancement in green and UV-LEDs has become the main concern of the lighting industry.

## 5. Factors Affecting SP–QW Coupling Enhancement in Nitride-Based LEDs

The main factors that affect the SP–QW coupling include penetration depth and metal nanostructure geometry. The penetration depth defines the p-GaN thickness and QW period number for which SP–QW coupling can be achieved successfully. The nanostructures of different metals and varying geometries can be used to increase the penetration depth. A dielectric layer between p-GaN and metal can also affect the penetration depth and SP–QW coupling enhancement in the emission wavelength range. In the following text, all these factors will be discussed and corelated on the basis of recent and the most relevant works in this field.

### 5.1. SP–QW Coupling Dependence on Penetration Depth and Capping Layer Thickness

One of the most important factors that affects the SP–QW coupling strength is the penetration depth of the SP fringing field in the p-GaN layer. The penetration depth has a limit beyond which SP–QW coupling enhancement cannot be achieved. It puts a limit on the p-GaN thickness through which the electric field has to penetrate for effective SP–QW coupling. The penetration depth (Z) of the SP fringing field into the GaN layer depends on the type of metal and wavelength (λ). It can be estimated by the following equation:

Z = λ/2π [(ε′_GaN_ − ε′_metal_)/ε′_metal_^2^]^1/2^ (1)

where ε′_GaN_ and ε′_metal_ are the real parts of the dielectric constants of GaN and metal, respectively. As the equation suggests, the penetration depth is defined by the values of the dielectric constants of the metal and material in contact with the metal layer (GaN); a dielectric layer of low dielectric constant sandwiched between metal and GaN can increase the penetration depth [73]. Different metals of varying dielectric constants can result in different penetration depths of the SP fringing field in GaN. K. Okamoto et al. calculated the penetration depths in GaN for the SP fringing fields produced by Al, Ag, and Au films as 77, 47, and 33 nm, respectively [10]. The penetration depth required for effective SP–QW coupling can be optimized by varying sizes, shapes, and periodicity of metal NPs that in turn affect the value of ε′_metal_. Metal gratings of varying ridge heights, widths, periods and shapes can also be used for the same purpose, whereas the metal films used for SP–QW coupling provide poor control over penetration depth optimization.

**Figure 2:**
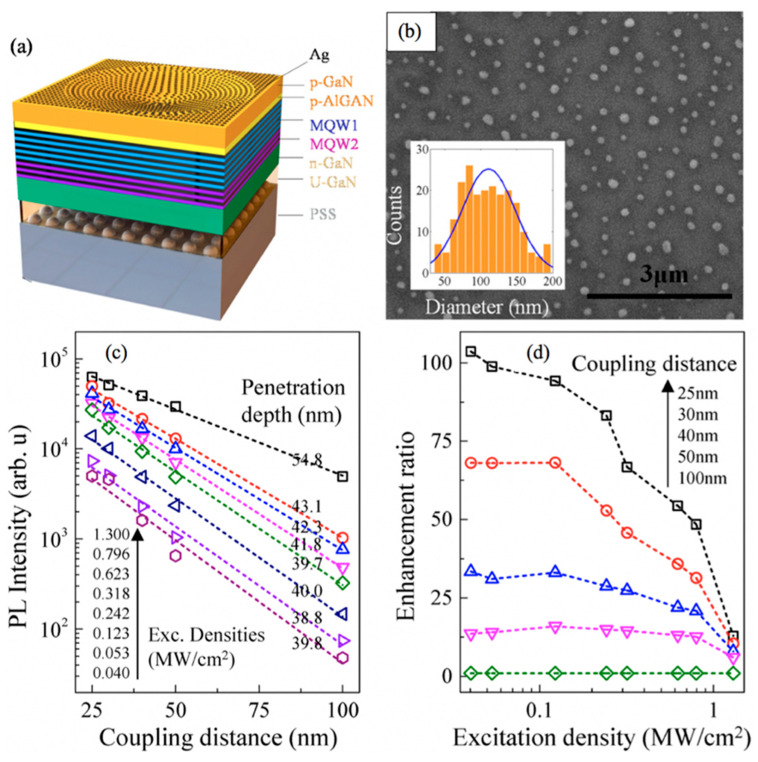
(**a**) Schematic diagram representing the basic structure of the SP-enhanced LED, (**b**) the plane-view SEM image of AgNPs inside the microcave, inset: diameter distribution of AgNPs, (**c**) the excitation power-dependent near-field PL vs. position of AgNPs in one of the SP-enhanced LEDs used in the study, and (**d**) the corresponding enhancement vs. excitation power of AgNPs for the same device at different coupling distances. Reproduced from [74].

Y. Li et al. reported the SP enhancement in dual-wavelength pseudo-micro blue LEDs that contained a microcave array of large aspect ratio on the p-GaN capping layer, as shown in Figure 2a [74]. The devices were fabricated on patterned sapphire substrates (PSS). AgNPs were formed in the microcave array patterned in p-GaN for SP–QW coupling, as shown in the SEM image in Figure 2b. They measured PL by using different laser power densities for devices with different coupling distances between p-GaN and AgNPs. The PL intensities and enhancement ratios dropped for increased coupling distances, as shown in Figure 2c,d. They observed an increase in penetration depths of the SP field in p-GaN with the increase in laser power densities but the enhancement ratios dropped because of the saturation of the density of states of SPs. The penetration depths varied between 39–55 nm for various excitation densities. They concluded that high excitation densities and shorter coupling distances (~25 nm) were suitable for PL enhancement.

W. F. Tse et al. studied the SP–QW coupling strength for various GaN capping layer thicknesses in blue and green SQW-LEDs with AgNPs on the top [17]. The green LEDs used in their studies gave high IQE enhancements for thicker p-GaN layers (~80 nm) compared with blue LEDs. The decline of IQE enhancement in green LEDs above 80 nm p-GaN thickness was attributed to the high intrinsic IQE (without SPs) of the LEDs with thicker p-GaN layers and consequently low relative IQE enhancements [67]. The IQE is usually high for the LEDs with relatively thick p-GaN layers due to the thermal annealing effect on QWs during the capping layer growth. Moreover, the electrical properties of an LED of thicker p-GaN are also superior. The SP enhancement of LEDs with thicker p-GaN is the major concern of the LED industry. However, the thermal annealing of QWs only at relatively lower temperatures can result in a higher IQE (less than 850 °C). The intrinsic IQE of an LED with a sufficiently thick p-GaN layer can also drop for high GaN growth temperatures due to the strong QCSE [24,48,75].

**Figure 3:**
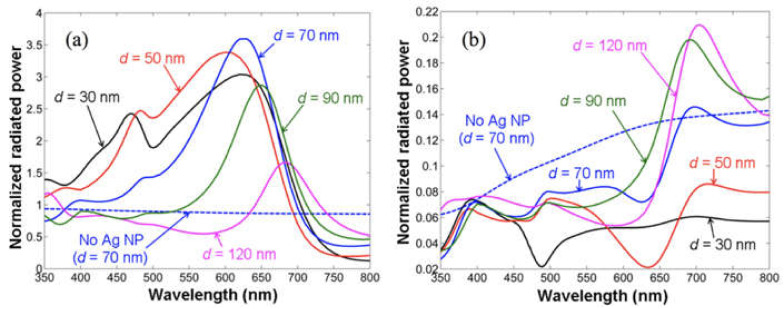
(**a**) Normalized downward and the (**b**) upward radiated powers of the NP–dipole coupling system as functions of wavelength for d = 30, 50, 70, 90, and 120 nm when the dipole is located exactly below the AgNP, i.e., at x = 0. The AgNP is surrounded by air in the upper-half-space. For comparison, the result in the case of no AgNP at the air/GaN interface with d = 70 nm is also plotted as the dashed curve. Reprinted with permission from [76]. Copyright 2018 The Optical Society.

In their study, the IQE of SP-enhanced blue SQW-LEDs dropped to an even smaller value than that of the intrinsic IQE for a coupling distance of ~100 nm. It indicates that the PL quenching phenomenon occurred instead of enhancement. The results were explained in terms of a redshift in the wavelength of the diminishing radiated power leaving a window in the original enhancement range proposed by Y. Kuo et al. [76]. They used the commercial COMSOL software to study the light emission and extraction in the presence of a radiating dipole coupled with the SP mode of an AgNP on GaN. The estimated upward and downward radiated powers are shown in Figure 3a,b, where the shift in downward radiated powers is more dramatic and strongly dependent on GaN thickness. For the enhanced SP–QW coupling, the radiated powers should lie in the emission wavelength range of the QWs. It otherwise results in a PL quenching phenomenon instead of enhancement. It should be noted that the redshift of radiated powers can suppress the IQE in blue LEDs and support the IQE in green LEDs. The upward radiated powers were high only in the wavelength ranges 600 nm and above. The results indicate that the downward radiated powers mainly define and support the SP–QW coupling. It is usually assumed that the SP resonance wavelength of a particular metal nanostructure geometry defines the SP–QW coupling strength. However, from this work, we conclude that the p-GaN thickness also plays an important role in the PL enhancement and quenching phenomena.

They pointed out that it was difficult to estimate the IQE dependence on p-GaN thickness since GaN growth involves heating that affects the QW properties. One possible workaround could be the heating of the reference LED to the p-GaN growth temperature for the same duration of time needed to grow a thicker p-GaN. This could partly help quantify the effect of heating on QWs for the true comparison of IQEs.

A. Fadil et al. fabricated SP-enhanced green LEDs by using Ag thin film on flat p-GaN and AgNPs in nano-hole patterned p-GaN [39]. They observed high PL intensity in the device with Ag film on a 130 nm flat p-GaN layer. The results are promising for SP enhancement without compromising the electrical properties of QW-LEDs by using sufficiently thick p-GaN. The results indicate that p-GaN patterning and using the metal nanostructures of different geometries do not necessarily support the emission enhancement. Their studies showed IQE enhancements by increasing the distance between the AgNPs embedded in the nano-holes patterned in p-GaN and QWs. This is contrary to the general expectations of SP enhancement with distance. They concluded that the metal nanostructure geometry was responsible for PL quenching at the shorter distance. Again, the PL enhancement and quenching for a particular metal geometry in their work can be explained by the results shown in Figure 3. The SP enhancement is a complex phenomenon that is influenced by many factors that contribute to SP–QW coupling and resonance. Not only the p-GaN thickness but the metal nanostructure geometry also has a leading role in deciding whether the PL will be enhanced or quenched. The energy coupled to LSP can be scattered out or dissipated in the metal nanostructures. The proportion of scattered to dissipated energy for a particular metal nanostructure decides whether the enhancement or quenching of PL will occur. Some metal nanostructures geometries support nonradiative absorption more than the radiative absorption [28,45,77]. The use of an appropriate metal of suitable nanostructure geometry on a p-GaN layer of right thickness can result in enhanced emission efficiencies.

Limited studies have been reported on SP enhancement of UV-LEDs due to the limited choice of metals that can support SP enhancement in the UV range. Al has been found to have SP resonance in the UV range needed for the SP enhancement of UV-LEDs; however, it is not easy to fabricate AlNPs. The penetration depth for the SP fringing field produced by Al can also be calculated in the UV range by using Equation (1). J. W. Lee et al. have recently reported the SP enhancement in an AlGaN MQW-LED using uniformly distributed AlNPs of average ~40 nm diameter obtained by block copolymer lithography [68]. By using the finite difference time domain (FDTD) simulation, they found that strong LSP resonance coupling could be achieved by using small AlNPs on ultrathin p-GaN. The SP-enhanced LEDs of 20 nm p-GaN thickness showed larger EL intensity and light output compared with reference LEDs. The Al NP-enhanced QW-LED gave a peak wavelength at 285 nm with an IQE enhancement of 57.5% and EL enhancement of 33.3% without significant degradation in electrical properties compared with reference LEDs. The results are promising for thinning the p-GaN layer for strong SP enhancement without compromising on the electrical properties of the devices.

In most of the studies reported on SP-enhanced QW LEDs, it is found that the IQE is enhanced only in devices with intrinsically lower IQE. C. H. Lin et al. reported the further enhancement of IQE for a commercial-quality blue LED, which has an intrinsic IQE higher than 80% through SP coupling [16]. As most of the SP-enhanced LEDs acquire a high IQE by sacrificing their electrical properties due to the requirement of small GaN thickness, the authors had already demonstrated in their previous works that Mg doping of the p-AlGaN electron blocking layer results in excellent electrical properties for even thinner p-GaN layers [24,48]. Mg doping works by reducing the hole potential barrier at EBL resulting in increased hole tunneling efficiency [78]. They reported a 90% IQE in SP-coupled and Mg-doped LED, which was 11% higher than that of the sample without SP coupling and Mg doping. The SP–QW coupling distance was as small as 66 nm. The AgNPs were fabricated on a low refractive index Ga-doped ZnO layer on the top to match the SP resonance wavelength with the emission wavelength of blue LED (~445 nm). They observed improvements in electrical characteristics of their devices by Mg doping that were not significantly degraded by SP coupling. The results demonstrate record high efficiencies for practical device applications and the commercialization of blue LEDs.

The use of an appropriate DI can also support penetration depth. J. He et al. observed an increase in LSPs penetration depth by using SiO_2_ DI between Al and MQW [73]. They observed a 2.6-fold deep UV enhancement of PL intensity and 2.3-fold IQE enhancement in their MQW structure. The results suggest that the dielectric properties of the DIs also affect the SP–QW coupling strength. The DIs of appropriate dielectric properties can support the penetration depth of the SP fringing field in the p-GaN.

### 5.2. SP–QW Coupling Dependence on QW Period Number

The SP coupling efficiency of an LED is also affected by the number of QWs (period number). W. F. Tse et al. studied the SP–QW coupling strength for various GaN capping layer thicknesses and MQW period numbers in blue and green QW-LEDs [17]. They observed a decreasing trend in the IQE above a critical period number for green MQW-LEDs that was explained in terms of poor SP–QW coupling between the deepest QW and AgNPs due to the large coupling distances.

They pointed out that it was difficult to estimate the IQE of an individual QW and quantify the sum of the individual IQEs in an MQW system precisely as the overgrowth process of a subsequent QW involves heating that influences the intrinsic IQE of an earlier-grown QW. The variation in strain in individual QWs being heated every time a QW is grown on the top leads to poor control on the efficiency evaluation of individual QWs. The earlier-grown QW can partly relax the compressive strain in subsequent QW layers. The QCSE is expected to reduce due to lower strain in the later-grown layers, leading to efficiency enhancement. However, we suggest that heating a single QW-LED (as a reference) to the QW growth temperature as many times as the period number of QWs could partly help quantify the influence of temperature on each QW in the MQW system.

The IQE enhancement was observed for a period number up to 5 for blue MQW-LEDs compared with green MQW-LEDs, which showed enhancement for a period number up to 4. The shifts in radiated powers shown in Figure 3 might also have some dependence on the QW period number. For blue LEDs, the SP enhancement for a QW period number higher than that of green LEDs indicates that the shifts in radiated powers were more supportive in the case of blue LEDs. Their results suggest that for a blue LED with a distance of more than 60 nm between the top QW and AgNPs, the optimized QW period number for effective SP coupling is 3–4 for a QW width of 3 and spacing of 12 nm. As the reported work demonstrates, the SP–QW coupling shows strong dependence on the QW period number, yet the topic is least explored and needs further investigation to develop better understanding.

### 5.3. SP–QW Coupling Dependence on the Metal and Metal Nanostructure Geometry

**Figure 4:**
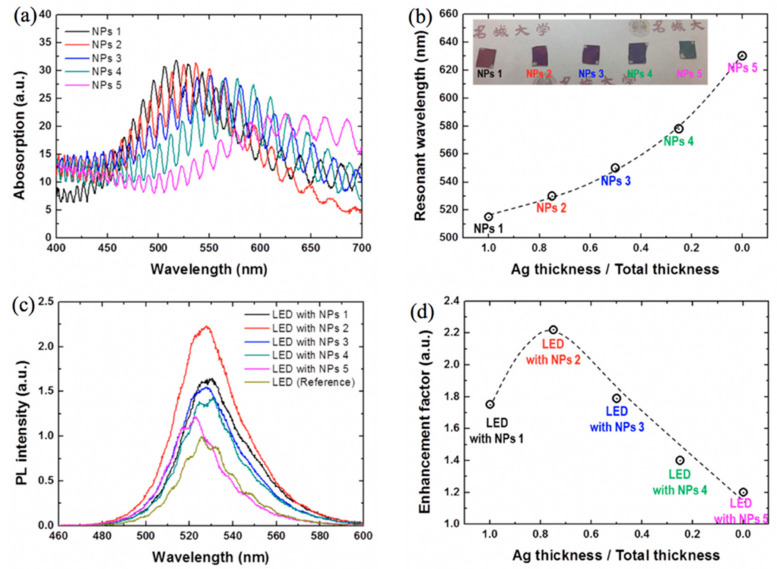
(**a**) Absorption spectra and (**b**) resonant wavelengths of the LSP modes for NPs of varying Au and Ag concentrations on the GaN layer. The inset shows the samples that have different colors due to different absorption properties. (**c**) PL spectra of the LEDs with and without NPs. (**d**) The enhancement factors of the LED with NPs. Reproduced from [27].

Enhanced SP–QW coupling can be achieved when the SP resonance mode of a metal resonates with the emission wavelength of the QW-LED. Different metals can give SP resonances in different wavelength ranges. The SP resonance wavelengths of most widely used Al, Ag, and Au thin films on GaN are centered at 243, 442, and 539 nm, respectively [27]. The SP resonance wavelength can be tuned to shift and match the QW emission wavelengths by using different metals of varying nanostructure geometries. The metal gratings as well as random and periodic nanostructures can be used for this purpose. Thin films, on the other hand, allow for poor control on resonance. The SP resonance wavelength can also be tuned by using double-metallic films or NPs [79]. Recently, R. Mano et al. achieved a high IQE in green LEDs by tuning the SP resonance wavelength [27]. They obtained double-metallic NPs by annealing Au/Ag multilayers of different thicknesses at 250 °C in N_2_, which allowed them to tune the SP resonance wavelength between 510 and 640 nm. Figure 4a shows the absorption spectra of the respective NPs that were fabricated by increasing the thicknesses of Au from 0 to 4 nm and decreasing the thicknesses of Ag from 4 to 0 nm. The peaks of the absorption spectra show the shift in the position of SP resonance wavelengths for different NPs. Figure 4b shows the change in the SP resonance wavelength for the ratio of Ag thicknesses to total thickness. The change in PL emission intensities was observed for NPs of different Ag and Au concentrations, as shown in Figure 4c. Figure 4d shows the change in enhancement factor with the change in the ratio of Ag thicknesses to total thickness.

**Figure 5:**
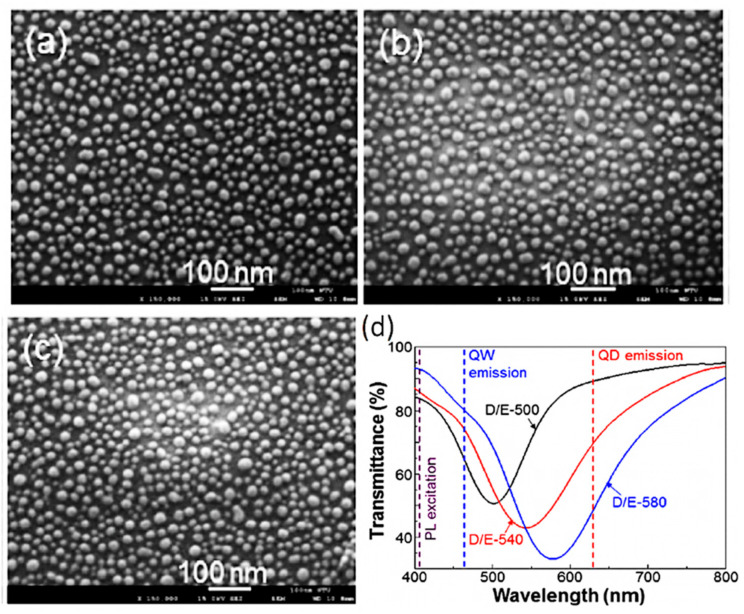
(**a**–**c**) SEM images of AgNPs for generating LSP resonances with peak wavelengths around 500, 540, and 580 nm, respectively. (**d**) Transmission spectra of the three AgNP structures shown in (**a**–**c**) illustrating the LSP resonances with peak wavelengths around 500, 540, and 580 nm. Reprinted with permission from [12]. Copyright 2018 The Optical Society.

C. H. Lin et al. experimentally demonstrated the shift in SP resonance wavelength by annealing the Ag films of varying thicknesses at different temperatures [12]. The purpose was to tune the SP resonance wavelength to achieve resonance with emission wavelengths of QW and quantum dots (QDs) used in their studies. The AgNPs’ sizes were found to increase slightly with Ag film thickness, as shown in Figure 5a–c. They observed a clear redshift in the position of SP resonance wavelength with the increase in AgNP size, as shown in Figure 5d. Three AgNP samples gave broad SP resonance wavelengths centered at 500, 540, and 580 nm. Since the AgNPs of random sizes and periodicity were fabricated, NPs of certain sizes and periodicity might result in sharp SP resonance wavelengths only covering a small wavelength range.

Generally, it is assumed that the SP coupling becomes stronger when the distance between the metal and QWs is decreased [8,10,52]. However, some metal nanostructures can give enhancement when the distance between the metal and QW is increased, depending on the metal nanostructure geometry. A. Fadil et al. observed emission enhancement with the increase in distance between AgNPs and QWs in green LEDs [39]. The distance between AgNPs and QWs was varied by embedding the AgNPs into micro- and nano-holes of varying depths in the p-GaN layer. Contrary to the expectations, the SP–QW coupling strength was found to show a direct proportion to the coupling distance in their device structures. They attributed the phenomenon to the nanostructure geometry of the metal–semiconductor interface that determines whether the resulting PL will be quenched or enhanced. They found that small AgNPs ~10–50 nm in the vicinity of large NPs quenched the PL emission, leading to weak SP–QW coupling in the green wavelength range. When the same device structure was modified by depositing Ag film on the surface, the PL enhanced instead of quenched. Here, we propose that the same phenomenon that leads to a redshift in the wavelength of diminishing radiated power described in Figure 3 might be responsible for the weak SP coupling for the given NP and p-GaN interface geometry [76]. From their reflectance spectroscopy data, they found that the large NPs surrounded by small NPs exhibit weak reflectance/high absorption. They suggest that high absorption leads to increased energy dissipation and PL quenching. They developed a similar understanding from their previous works [54,80].

**Figure 6:**
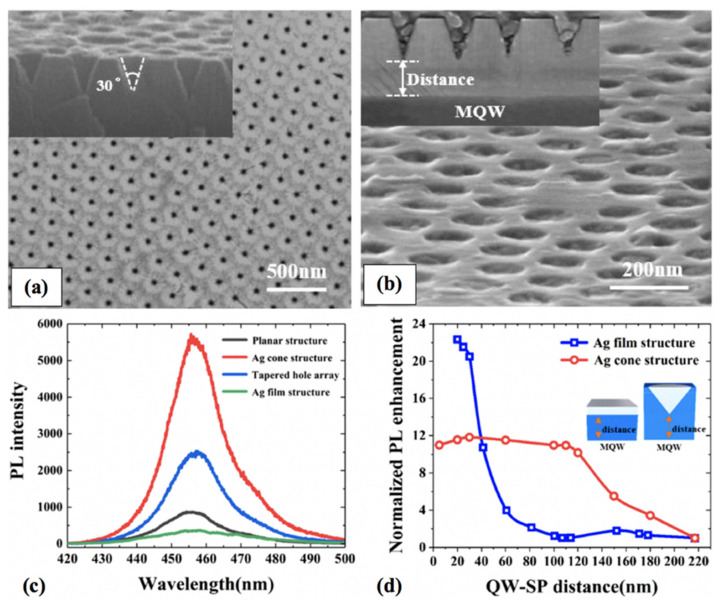
(**a**) Nano-cones on the p-GaN surface and (**b**) embedded tapered Ag; the inset images are side view of nano-cones on the p-GaN surface and embedded tapered Ag, respectively. (**c**) PL spectra of cone and plane structures with and without Ag at a coupling distance of 100 nm. (**d**) Normalized PL enhancement of LEDs with Ag cone and film as a function of coupling distance. The inset shows the definition of the QW-SP distance for different structures. Reprinted with permission from [83]. Copyright 2020 The Optical Society.

For blue GaN-based LEDs, the coupling distance less than 42 nm has been reported to be appropriate for effective SP–QW coupling when using Ag metal that is far less than the GaN thickness required (>70 nm) to achieve reasonable electrical performance [10,81,82]. However, proper engineering of the metal nanostructures can allow SP–QW coupling through thick p-GaN layers. Recently, Xuzheng Wang et al. have demonstrated that a tapered Ag array structure on a heavily textured p-GaN layer resulted in an increased coupling efficiency for coupling distance far beyond the penetration depth of a blue LED [83]. The SEM surface images of the patterned surface are shown in Figure 6a,b. They explained that the metal conical tip accumulates the SP energy and the corrugated surface provides the missing momentum in such a structure. The structure resulted in a 16-fold increase in electroluminescence intensity compared with a planar LED for 100 nm coupling distance. The work was inspired by the previous studies on tapered waveguides which conclude that the giant electric fields can be produced using accumulated SP energy at metal conical tips that have much slower distance-dependent decay rates [84,85]. The tapered hole array structures with Ag showed the highest PL enhancement as the high density of states at the metal tip provided strong LSP energy even at distances as large as 100 nm, as shown in Figure 6c. The results are promising to avoid the short coupling distance requirement for blue LEDs by designing the Ag nanostructures. Figure 6d shows the distance dependence of normalized PL for a planar Ag LED structure compared with a tapered Ag structure. Interestingly, the Ag cone structure gave lower PL enhancement for distances ≤ 40 nm compared with the planar Ag film structure. This indicates that the PL quenching phenomenon occurred when the metal cone structure was at shorter distances from QW. The coupling efficiency of the Ag cone structure remained almost constant for distances up to 100 nm and dropped afterward, compared with the Ag film structure, which showed PL enhancement efficiency only for thicknesses up to 40 nm, far less than the required GaN thickness to ensure high electrical performance. The tapered Ag cone structure resulted in enhanced EL intensities, but the threshold voltage was slightly higher. Although the structure seems promising for a relatively thick p-GaN layer for practical blue LED application, the high cost of lithography and design complications are serious limitations for such structures.

The role of LSPs in LEDs that can be excited by using nanostructured metal films can be compared with SPPs that are excited on relatively smooth and continuous metal films. Both the LSPs and SPPs are found to contribute significantly to emission enhancement [86]. Grating structures are very powerful among different metal nanostructures for their ability to enhance both the SPP and LSP modes for SP–QW coupling. The counter-propagating SPPs can result in a standing wave-like feature in a grating system through interference that forms a localized resonance feature similar to LSPs. Similarly, the metal films of nano-scale roughness can also excite LSPs. Therefore, it is difficult to compare the effectiveness of SPPs and LSPs in the emission enhancement of LEDs. A standing wave-like mode can, however, cover a large coupling range in lateral dimension compared with that of a strongly localized behavior of an LSP mode. The effective coupling distance between the QW and metal can be reduced by using gratings when the ridge tips are facing downwards. The p-GaN layer can be kept thicker between the ridges in such devices to preserve the electrical characteristics [48]. It is of profound importance to explore the ridge height, width, period, and shape dependence of SPP/LSP modes in gratings of various metals to understand the role of SPPs and LSPs in SP–QW coupling enhancement.

**Figure 7:**
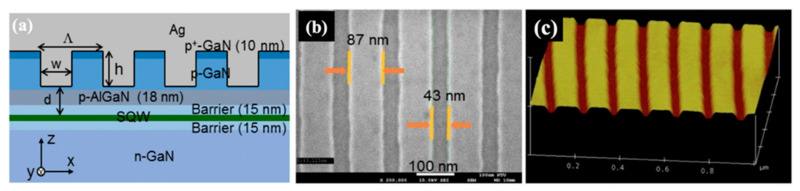
(**a**) Schematic demonstration of an LED structure with an Ag grating on the top. (**b**) Plan-view SEM and (**c**) tilted AFM images, respectively, of the grating structure before Ag deposition in one of the LEDs. Reprinted with permission from [48]. Copyright 2018 The Optical Society.

Y. F. Yao et al. studied the Ag grating height and width dependence of the preferred SP–QW coupling mode among SPPs and LSPs [48]. The ridge height defines the strength of SPP and LSP resonances. The small (large) ridge heights result in strong SPPs (LSPs). By theoretical calculations, they found the grating period of 130 nm to be suitable for strong SPP coupling in green SQW-LEDs. They fabricated SQW-LEDs of various p-GaN thicknesses while keeping the grating period fixed at Λ = 130 nm as shown in Figure 7a. They used polarization-dependent reflection spectra to understand the SP resonance behaviors of Ag grating structures. The LEDs without grating structures were used as references for comparison. Most of the works suggest that the larger p-GaN thickness is better to obtain increased emission efficiencies. However, in their work, the IQE was reported to decrease with p-GaN thickness for the reference LEDs. The effect was attributed to the longer heating duration for thicker p-GaN growth at 970 °C that resulted in the degradation of the QW layer. Temperatures above 900 °C have been reported to be enough for changing the IQE of an InGaN/GaN QW structure [24,75].

They found that LSP coupling dominates for larger grating ridge height. The device structure that they theoretically designed to compensate the momentum mismatch for effective SPP coupling also gave stronger LSP coupling compared with SPP. Mg pre-flow process was applied, that has been proved useful in preserving the electrical properties of LEDs, even for thinner GaN layers [24,78]. The effective QW depths from the top of the epitaxial structure varied between 53–93 nm. The plan-view SEM and tilted AFM images of the grating structure before Ag deposition are shown in Figure 7b,c, respectively. They reported a 6–8% enhancement of IQE with grating structures compared with reference LEDs without grating structures. With Ag deposition on grating structures, the IQEs higher than 30% were achieved due to the reduction in PL decay time. Resistance was found to increase with p-GaN thickness and fabrication of the Ag grating structure on the top. The efficiency droop range reduced with SP coupling. They concluded that the IQE and EL enhancements are higher for devices with LSP-dominated coupling compared with those of SPP-dominated coupling. The grating structures of very complex geometries have also been reported by several authors, but the fabrication cost increases with complexity [48]. Moreover, despite the high cost and complications, the enhancement in efficiencies is not very significant.

### 5.4. SP–QW Coupling Dependence on Dielectric Interlayer

**Figure 8:**
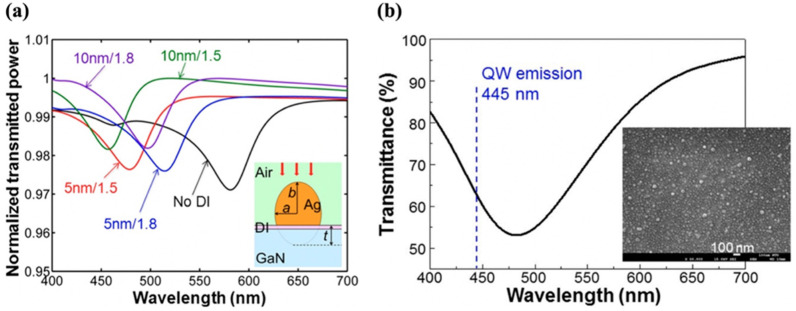
(**a**) Simulated normalized transmission spectra of an AgNP with various DI conditions, including the case of no DI and the cases of DI thicknesses at 5 and 10 nm and refractive indices at 1.5 and 1.8. The insert shows the geometry of simulation, including the definitions of parameters a, b, and t. Reproduced with permission from [52]. Copyright 2014 AIP Publishing. (**b**) Transmission spectrum of the AgNPs on a GaZnO/u-GaN template. The vertical dashed line indicates the QW emission wavelength at 445 nm. The inset shows the SEM image of the surface AgNPs fabricated on the GaZnO layer. Reprinted with permission from [16]. Copyright 2018 The Optical Society.

The SP resonance wavelength of metal can be adjusted to resonate with the emission wavelength of a QW-LED for effective SP–QW coupling by using an appropriate dielectric interlayer (DI) between the metal and p-GaN layer. While the SP resonance wavelength can also be controlled by the geometry of the metal nanostructures, using a low refractive index layer under the metal can give an easier control on resonance. Enhanced emission has been reported by using a low refractive index SiO_2_ DI between the metal and GaN [87,88]. C. H. Lin et al. studied the effect of SiO_2_ DIs of varying thicknesses between Ag and GaN on the SP resonance wavelength by simulation as shown in Figure 8a [52]. In their recent work, they used a low refractive index Ga-doped ZnO layer between AgNPs and GaN to achieve SP resonance in the blue range [16]. They kept the AgNP size sufficiently small to achieve better resonance. The peak of SP resonance wavelength centered at 482.2 nm was in close resonance but not well aligned with the QW emission wavelength, as shown in Figure 8b. The structure resulted in a record IQE of 90%. The emission efficiency above 90% can be expected by further optimization of the metal geometry or replacing the dielectric interlayer with a layer of an even lower refractive index compared with that of Ga-doped ZnO.

The larger AgNPs on a relatively high refractive index GaN surface result in the redshift of the LSP resonance wavelength. W. F. Tse et al. blue-shifted the position of the SP resonance wavelength of the large AgNPs by using a low refractive index SiO_2_ between AgNPs and p-GaN to obtain enhanced blue emission from a QW-LED [17].

Not only does it affect the position of an SP resonance wavelength, but the use of a DI is also reported to affect the penetration depth of the SP fringing field. J. He et al. reported a 2.6-fold deep UV emission enhancement of PL intensity and 2.3-fold IQE enhancement in MQWs by using AlNPs on SiO_2_ DI [73]. They suggest that the presence of a SiO_2_ DI resulted in the increased penetration depth of LSPs. A dielectric layer between QWs and LSPs is also found to lower the EQE droop in QW-LEDs and increase the LEE [52,53].

## 6. Conclusions and Outlook

The SP–QW coupling depends on the penetration depth of the SP fringing field in the p-GaN and the extent of overlap between the SP resonance wavelength of the metal and the QW emission wavelength. The penetration depth in turn defines the p-GaN thickness and the maximum QW period number for which SP enhancement can be achieved. The penetration depth in the required wavelength range varies for different metal films, nanostructures, and gratings. When the thickness of p-GaN is below the penetration depth and the SP resonance wavelength of the metal on the GaN structure coincides with the QW emission wavelength, the SP–QW coupling is enhanced several folds. The penetration depth and the resonance of SP wavelength of metal with QW emission wavelength can be controlled by varying the metal nanostructure geometry and using dielectric layers between GaN and the metal. The small p-GaN thickness and increased QW period number does not necessarily lead to high IQE enhancement in SP-enhanced QW-LEDs. The SP enhancement is a complex phenomenon that depends on the strength of the resulting radiated powers in the required wavelength range. Since blue LEDs have already achieved high efficiencies, the green and UV-LEDs require more attention. New metals and metal alloys of varying geometries on different DIs should be explored for the possible enhancement of green and UV LEDs.

## Data Availability

No new data were created or analyzed in this study. Data sharing is not applicable to this article.
